# THE ROLE OF ENGAGEMENT IN SUPPORTING AND LEVERAGING GERONTOLOGY AND GERIATRIC PROGRAMS

**DOI:** 10.1093/geroni/igz038.2284

**Published:** 2019-11-08

**Authors:** Christine Fruhauf

**Affiliations:** Colorado State University, Fort Collins, Colorado, United States

## Abstract

For nearly 20 years, gerontology and geriatric administrators and faculty have been challenged by managing “tough times” related to low enrollment and reduced or limited funding for their programs. At the same time, the aging population continues to increase and the need for highly trained individuals to work with and on behalf of older adults are needed in all sectors of the workforce. In this paper, I will build on previous empirical and theoretical work from AGHE and GSA Fellows as I integrate personal experience from my fifteen years at a land-grant university, whereby I serve as the coordinator of our undergraduate gerontology minor. In particular, in an effort to uplift the 2019 conference theme, I will organize my thoughts from the lens of university-community engagement, as I describe how to best harness networks to support and leverage gerontology and geriatrics programs.

